# Induction of C/EBP homologous protein-mediated apoptosis and autophagy by licochalcone A in non-small cell lung cancer cells

**DOI:** 10.1038/srep26241

**Published:** 2016-05-17

**Authors:** Zheng-Hai Tang, Xin Chen, Zhao-Yu Wang, Ke Chai, Ya-Fang Wang, Xiao-Huang Xu, Xiao-Wen Wang, Jia-Hong Lu, Yi-Tao Wang, Xiu-Ping Chen, Jin-Jian Lu

**Affiliations:** 1 State Key Laboratory of Quality Research in Chinese Medicine, Institute of Chinese Medical Sciences, University of Macau, Macao, China; 2 Medical Center, Yuquan Hospital, Tsinghua University, Beijing, China

## Abstract

Licochalcone A (LCA), a flavonoid isolated from the famous Chinese medicinal herb *Glycyrrhiza uralensis* Fisch, presents obvious anti-cancer effects. In this study, the anti-cancer effects and potential mechanisms of LCA in non-small cell lung cancer (NSCLC) cells were studied. LCA decreased cell viability, increased lactate dehydrogenase release, and induced apoptosis in a concentration-dependent manner in NSCLC cells while not in human embryonic lung fibroblast cells. The expression of phosphatidylethanolamine-modified microtubule-associated protein light-chain 3 (LC3-II) and formation of GFP-LC3 punta, two autophagic markers, were increased after treatment with LCA. LCA-induced LC3-II expression was increased when combined with chloroquine (CQ), while knock-down of autophagy related protein (ATG) 7 or ATG5 reversed LCA-induced LC3-II expression and GFP-LC3 punta formation, suggesting that LCA induced autophagy in NSCLC cells. Inhibition of autophagy could not reverse the LCA-induced cell viability decrease and apoptosis. In addition, LCA increased the expression of endoplasmic reticulum stress related proteins, such as binding immunoglobulin protein and C/EBP homologous protein (CHOP). Knock-down of CHOP reversed LCA-induced cell viability decrease, apoptosis, and autophagy. Taken together, LCA-induced autophagic effect is an accompanied phenomenon in NSCLC cells, and CHOP is critical for LCA-induced cell viability decrease, apoptosis, and autophagy.

Non-small cell lung cancer (NSCLC) is one of the most frequently diagnosed cancers and the leading cause of cancer death worldwide, contributing to more than one-quarter of all cancer deaths^1^,^2^. Most of NSCLC patients present with advanced disease upon diagnosis and the therapeutic strategy for these patients is drug therapy^3^. The survival rate of NSCLC patients is significantly increased under precision medicine guidance, for example, epidermal growth factor receptor tyrosine kinase inhibitors (EGFR-TKI) (erlotinib, gefitinib, and afatinib) have been successfully utilized in NSCLC patients with EGFR sensitive mutation^4^,^5^. Meanwhile, more than half of NSCLC patients are harboring wild type EGFR for whom the treatment strategies are cisplatin- or docetaxel-based chemotherapy^6^. Due to the obvious drug resistance and severe side effects of cisplatin and docetaxel, the seeking of novel chemotherapeutics and chemical scaffolds of chemotherapeutics for NSCLC patients with wild type EGFR is necessary^7^,^8^.

Natural products are a large reservoir for anti-cancer drug discovery due to their enormous structural diversity. Many anti-cancer agents, such as paclitaxel, vincristine, and etoposide, are naturally-derived and play critical roles in chemotherapy^9^,^10^. Licochalcone A (LCA), one of the main active flavonoids isolated from the famous Chinese medicinal herb *Glycyrrhiza uralensis* Fisch, presents a wide range of pharmacological effects, such as anti-cancer^11^, anti-inflammation^12^, and anti-osteoporosis^13^. The anti-cancer effect of LCA has been demonstrated in diverse types of cancer cells, including gastric cancer BGC-823 cells^11^, hepatocellular carcinoma HepG2 cells^14^, as well as ovarian cancer OVCAR-3 and SK-OV-3 cells^15^. Several *in vivo* studies indicated that LCA presents remarkable therapeutic effects for gastric cancer^11^, cervical cancer^16^, and colon cacner^17^,^18^,^19^. Moreover, LCA obviously inhibited the cisplatin-induced kidney damage without affecting its anti-cancer effects^20^. Reducing cell viability, inducing apoptosis and cell cycle arrest, as well as inhibiting cell metastasis and angiogenesis were reported to be the mechanisms for its anti-cancer activity^11^,^18^,^21^.

Autophagy is a conserved cellular degradation system that is responsible for degrading and recycling damaged or unnecessary cytoplasmic contents in a lysosome-dependent manner^22^. The process begins when phagophores emerge and nucleate at the phagophore assembly site. Then, the phagosomes elongate to form autophagosomes via the ubiquitination-like systems. The autophagosomes then fuse with lysosomes to form autolysosomes and the autolysosomes degrade their cargos^23^. Previous studies indicated that a mass of compounds could induce autophagy for cell survival or result in cell death by various mechanisms, *e.g.* glycyrrhetinic acid induces cytoprotective autophagy in NSCLC via the inositol-requiring enzyme 1α - c-Jun N-terminal kinase cascade, while clioquinol increased autophagic cell death in leukemia and myeloma cells by inhibition of mTOR cascade^24^,^25^.

In the present study, the effects of LCA in EGFR wild type NSCLC A549 and NCI-H1299 cells in terms of cell viability, apoptosis, and autophagy were evaluated. Furthermore, the potential mechanisms for LCA-induced apoptosis and autophagy were studied.

## Results

### LCA decreased cell viability and increased lactate dehydrogenase (LDH) release in NSCLC cells while not in normal cells

**Figure 1:**
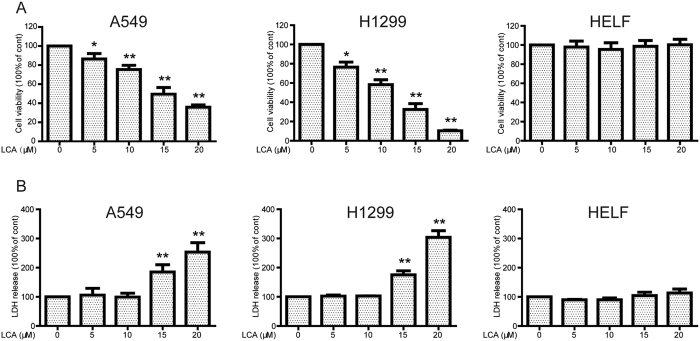
LCA decreased cell viability and increased LDH release in NSCLC cells while not in normal cells. **(A)** A549, NCI-H1299, and HELF cells were treated with indicated concentrations of LCA for 24 h. The cell viability was evaluated by MTT assay. *P < 0.05, **P < 0.01, compared with 0 μM LCA treatment. **(B)** Cells were treated with 0, 5, 10, 15, and 20 μM LCA for 24 h, and the LDH release was determined according to the manufacturer’s introductions. *P < 0.05 and **P < 0.01, compared with 0 μM LCA treatment.

First, the effects of LCA on cell viability were evaluated by 3-(4,5-dimethylthiazol-2-yl)-2, 5-Diphenyltetrazolium bromide (MTT) assay. As shown in Fig. 1A, the cell viability of A549 and NCI-H1299 were significantly decreased in a concentration-dependent manner after incubation with LCA for 24 h (cell viabilities were 86.40%, 75.30%, 49.50%, and 35.80% in A549 cells and 76.45%, 58.27%, 32.56%, and 10.40% in NCI-H1299 cells after treatment with 5, 10, 15, and 20 μM LCA, respectively). The LDH, released to the culture medium, was increased after treatment with LCA in both A549 (LDH released to the culture medium was 185.26% and 253.46% in 15 and 20 μM LCA-treated groups, respectively) and NCI-H1299 cell lines (LDH released to the culture medium was 175.20% and 303.85% in 15 and 20 μM LCA-treated groups, respectively) (Fig. 1B). In addition, LCA did not affect the cell viability and LDH release in normal human embryonic lung fibroblast (HELF) cells (Fig. 1) and human liver LO2 cells (Fig. S1). The aforementioned results suggested that LCA preferentially presented cytotoxicity in cancer cells.

### LCA induced apoptosis in NSCLC cells while not in normal cells

**Figure 2:**
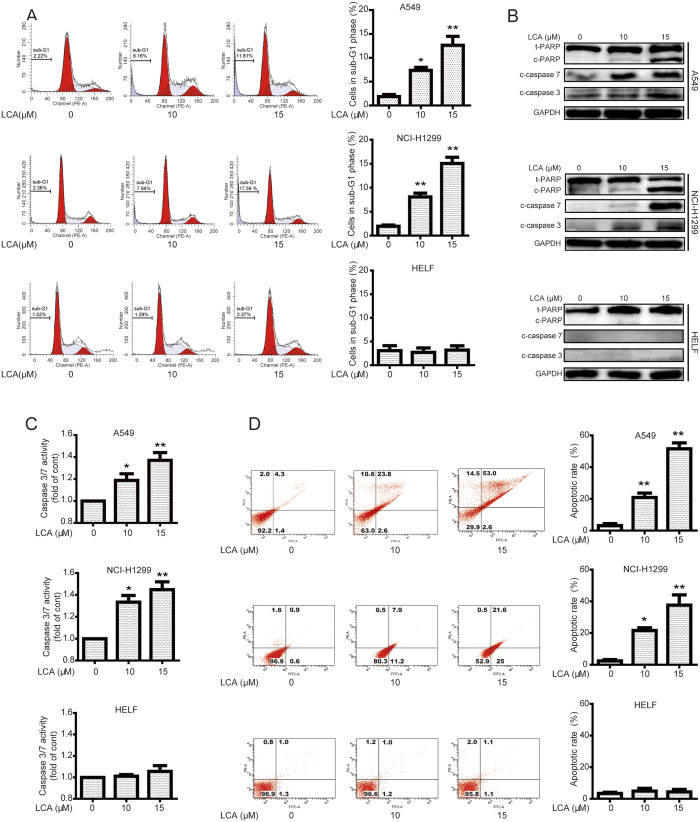
LCA induced apoptosis in NSCLC cells while not in normal cells. **(A)** Cells were treated with indicated concentrations of LCA for 24 h. The cells in sub-G1 phase were evaluated by flow cytometry. *P < 0.05 and **P < 0.01. **(B)** After treatment with LCA for 24 h, cells were analyzed to determine indicated changes of proteins by western blot analysis. The blots were run under the same conditions and the full-length blots were shown in supplementary materials. **(C)** Cells were treated with LCA for 24 h, and the activation of caspase 3/7 was determined using a commercial kit. *P < 0.05 and **P < 0.01. **(D)** After treatment with LCA for 24 h, apoptotic cells were stained with annexin V/PI and analyzed by a flow cytometry. *P < 0.05 and **P < 0.01.

To investigate the underlying mechanism of LCA-decreased cell viability, the apoptotic effect of LCA in NSCLC cells was examined by various methods. As shown in Fig. 2A, cells in sub-G1 phase were increased after incubation with LCA in A549 (1.87%, 7.36%, and 12.63% for 0, 10, and 15 μM LCA treatment, respectively) and NCI-H1299 (1.96%, 8.08%, and 15.06% for 0, 10, and 15 μM LCA treatment, respectively) cells. However, the cell numbers of sub-G1 phase were not obviously increased in LCA-treated HELF cells (3.08%, 2.72%, and 3.19% for 0, 10, and 15 μM LCA treatment, respectively) (Fig. 2A). The protein expression of cleaved poly (ADP-ribose) polymerase (c-PARP), cleaved-caspase 7 (c-caspase 7), and cleaved-caspase 3 (c-caspase 3), which are biomarkers of apoptosis^26^, were all remarkably increased after treatment with LCA in NSCLC cells, while not in HELF cells (Fig. 2B). In addition, the activation of caspase 3/7 activity was remarkably increased after 10 or 15 μM of LCA treatment in the NSCLC cells but not in HELF cells (Fig. 2C). Furthermore, annexin V-FITC and propidium iodide (PI) dual labeling assay indicated that exposure of NSCLC cells to LCA increased apoptotic cell percentages from 3.2% (0 μM) to 20.9% (10 μM) and 51.9% (15 μM) in A549 cells, and from 2.4% (0 μM) to 21.6% (10 μM) and 37.6% (15 μM) in NCI-H1299 cells, while the apoptotic cells were not increased after LCA treatment in HELF cells (Fig. 2D). Collectively, these data suggested that LCA induced apoptosis in NSCLC cells while not in normal lung cells.

### LCA induced autophagy in NSCLC cells

**Figure 3:**
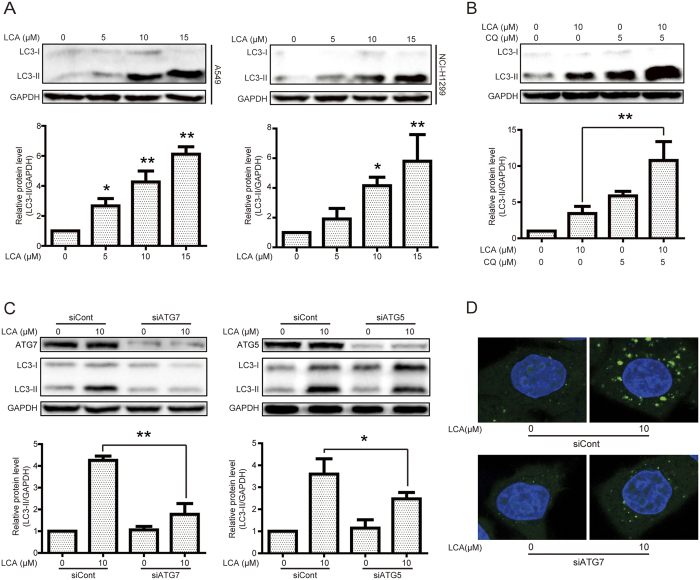
LCA induced autophagy in NSCLC cells. **(A)** A549 and NCI-H1299 cells were treated with various concentrations of LCA for 24 h, and cell extracts were analyzed to determine the changes of protein expression by western blot analysis. *P < 0.05 and **P < 0.01. The blots were run under the same conditions and the full-length blots were shown in supplementary materials. **(B)** A549 cells were cultured in 10 μM LCA for 24 h with or without CQ pretreatment (5 μM, 1 h). Cell extracts were analyzed for protein expression using western blot analysis. *P < 0.05 and **P < 0.01. **(C)** After transient transfection with scramble, ATG7, or ATG5 siRNA for 24 h, A549 cells were treated with 10 μM LCA for 24 h. The indicated protein expression was evaluated by western blot analysis. *P < 0.05 and **P < 0.01. **(D)** A549 cells were transiently transfected with GFP-LC3 plasmid for 24 h and ATG7 siRNA for 24 h. LCA (10 μM) was added into the cells, and cultured for another 24 h. GFP-LC3 punta were examined using the confocal microscope.

We then determined whether or not LCA could induce autophagy in NSCLC A549 and NCI-H1299 cells. First, western blot assay was performed to examine the protein expression of LC3-II, which is essential for autophagy formation and mainly used as a protein marker of this phenomenon^27^. LCA significantly increased LC3-II expression in a concentration-dependent manner in A549 and NCI-H1299 cells (Fig. 3A) while not in HELF cells (Fig. S2). Combination treatment using LCA and the autophagy inhibitor chloroquine (CQ), which disrupts the function of lysosome to inhibit autophagy^28^, exhibited more LC3-II expression than that of LCA or CQ treatment alone (Fig. 3B). The autophagy related protein (ATG)7 and ATG5, which activate the LC3-II formation to promote expansion of the phagophore membrane and recruit cargos to autophagosome, play critical roles in the process of autophagy^23^. Herein, LCA-induced LC3-II was decreased after silence of ATG7 or ATG5 (Fig. 3C). Besides, the formation of GFP-LC3 punta is another widely used autophagy process biomarker^28^. After transiently transfected GFP-LC3 plasmid into A549 cells, LCA obviously induced GFP-LC3 punta formation and silence of ATG7 decreased LCA-induced GFP-LC3 punta formation (Fig. 3D). In addition, LCA-induced GFP-LC3 punta formation was further confirmed in HeLa cells with a GFP-LC3 stable expression and knock-down of ATG7 also remarkably reversed LCA-induced GFP-LC3 punta formation in these cells (Fig. S3).

### LCA-induced cell viability and apoptosis were not changed after inhibition of autophagy

**Figure 4:**
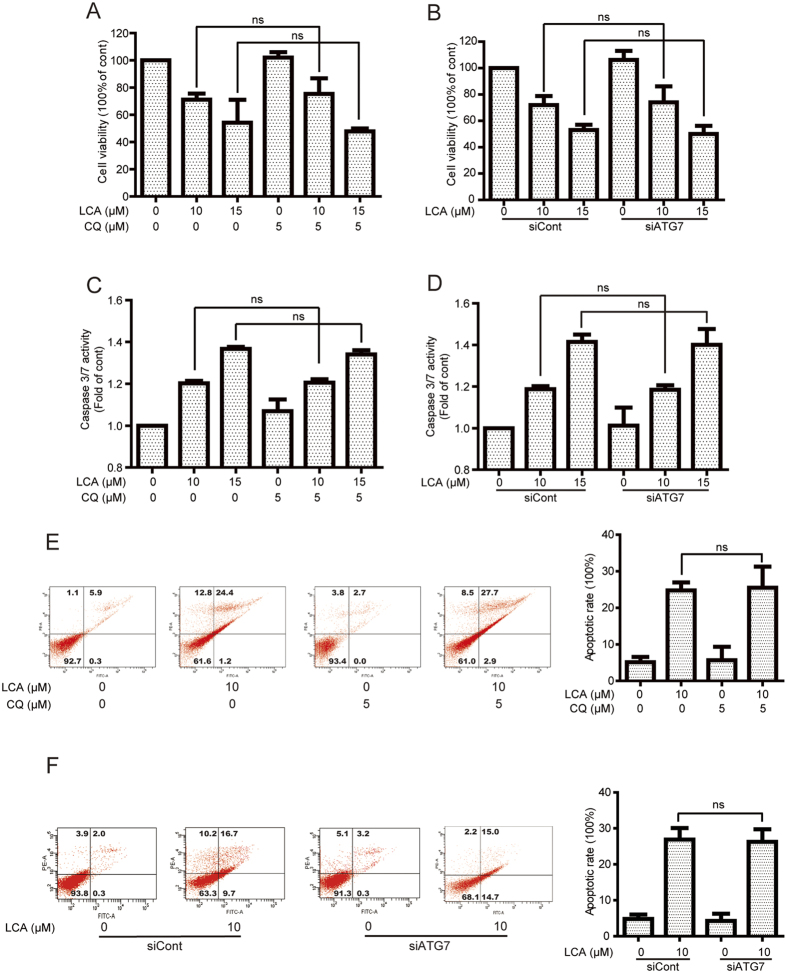
LCA-induced cell viability and apoptosis were not altered by inhibition of autophagy. **(A,B)** A549 cells were treated with various concentrations of LCA for 24 h with or without pretreatment with CQ (10 μM, 1 h) or ATG7 siRNA. Cell viability was then evaluated using MTT assay. **(C,D)** After inhibition of autophagy by pretreatment of CQ or silence of ATG7, cells were treated with 10 and 15 μM LCA for 24 h. Caspase 3/7 activation was detected using a commercial kit. **(E,F)** After treatment with 10 μM LCA for 24 h with or without pretreatment of CQ or ATG7 siRNA, cells were stained with annexin V/PI and analyzed by a flow cytometry. The “ns” means “no statistical difference”.

The effects of autophagy (pro-survival or pro-death) in cancer therapy remain complex and inconclusive^29^. In the present study, LCA-induced cell viability decrease and apoptosis were evaluated after pharmacological and genetic inhibition of autophagy. As shown in Fig. 4A, pretreatment of the autophagy inhibitor CQ (5 μM, 1 h) could not statistically affect LCA-induced cell viability in A549 cells (cell viabilities for 10 and 15 μM LCA-treated cells were changed from 71.18% and 54.26% to 75.34% and 47.81% when pretreatment with CQ, respectively). The cell viability of LCA was also not statistically altered after knock-down of ATG7, *e.g.* cell viabilities were changed from 71.92% (10 μM LCA) and 53.03% (15 μM LCA) to 74.06% (10 μM LCA + siATG7) and 50.04% (15 μM LCA + siATG7), respectively (Fig. 4B). In addition, the apoptotic effect of LCA in NSCLC cells was determined after inhibition of autophagy. Firstly, the activation of caspase 3/7, which is responsible for the execution of apoptosis, was detected^26^. As shown in Fig. 4C,D, there was no significantly change in LCA-induced caspase 3/7 activities after autophagy inhibition. The annexin V-FITC and PI dual labeling further confirmed that inhibition of autophagy has no significant effect on LCA-induced apoptosis. The apoptotic rate of LCA-treated A549 cells was 24.76% in 10 μM LCA and 25.53% in 10 μM LCA with 5 μM CQ, and 26.90% in 10 μM LCA and 26.23% in 10 μM LCA with siATG7 (Fig. 4E,F).

### LCA induced apoptosis and autophagy in a CHOP-dependent manner

**Figure 5:**
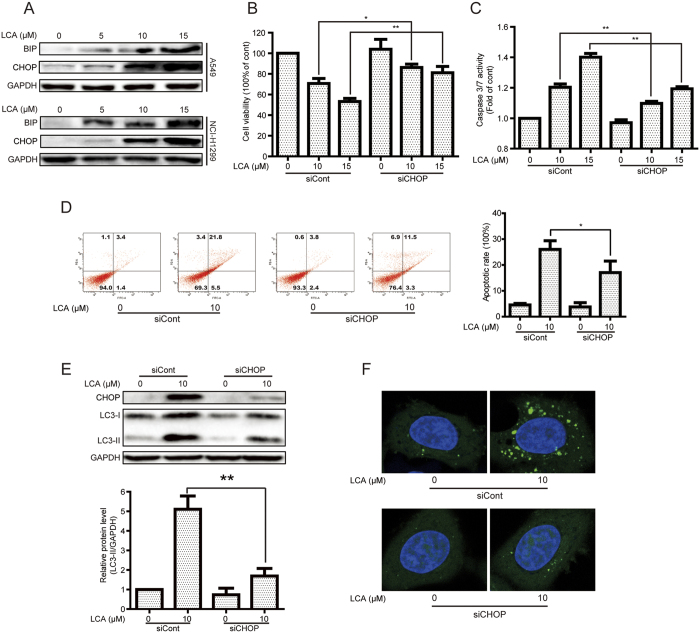
LCA induced apoptosis and autophagy in a CHOP-dependent manner. **(A)** A549 and NCI-H1299 cells were treated with indicated concentrations of LCA for 24 h, and the expression of ER stress related proteins was evaluated by western blot analysis. The blots were run under the same conditions and the full-length blots were shown in supplementary materials. **(B–D)** After knock-down of CHOP by siRNA, A549 cells were treated with LCA for 24 h. The cell viabilities were evaluated by MTT assay, caspase 3/7 activation was detected using a commercial kit, and apoptotic cells were studied by annexin V/PI staining, respectively. *P < 0.05 and **P < 0.01. **(E)** After transient transfected with CHOP siRNA for 24 h, A549 cells were treated with 10 μM LCA for 24 h. The indicated protein expression was evaluated by western blot analysis. *P < 0.05 and **P < 0.01. **(F)** A549 cells were transiently transfected with GFP-LC3 plasmid for 24 h and CHOP siRNA for 24 h. Cells were then treated with 10 μM LCA for 24 h and the GFP-LC3 punta formation was examined using a confocal microscope.

Apoptosis and autophagy can be induced by activating endoplasmic reticulum (ER) stress^30^,^31^. Herein, we indicated that LCA induced ER stress in A549 and NCI-H1299 cells as demonstrated by increasing the expression of ER stress protein markers, such as binding immunoglobulin protein (BIP) and C/EBP homologous protein (CHOP) (Fig. 5A). We then detected whether LCA-induced cell viability decrease and apoptosis were dependent on CHOP activation. As shown in Fig. 5B, LCA-decreased cell viability was reversed after silence of CHOP (cell viabilities changed from 70.73% (10 μM LCA) and 53.21% (15 μM LCA) to 86.23% (10 μM LCA + siCHOP) and 81.15% (15 μM LCA + siCHOP), respectively. The LCA-induced apoptotic effect was studied as well after silence of CHOP. LCA-induced caspase 3/7 activation was decreased after CHOP siRNA (Fig. 5C) and LCA-induced cell apoptosis could be reversed after knock-down of CHOP, *e.g.* apoptotic cells were changed from 26.07% (10 μM LCA) to 17.07% (10 μM LCA + siCHOP) (Fig. 5D). In addition, we detected whether LCA-induced autophagy was dependent on CHOP. As shown in Fig. 5E,F, silence of CHOP by siRNA obviously decreased LCA-induced LC3-II expression and GFP-LC3 punta formation in NSCLC cells. Besides, the LCA-induced autophagy by CHOP activation was further confirmed in HeLa cells with GFP-LC3 stable expression (Fig. S4). Collectively, CHOP plays a critical role in both LCA-induced apoptosis and autophagy.

## Discussion

Most of NSCLC patients are harboring wild type EGFR and these patients could not benefit from EGFR-TKI therapy^32^. The chemotherapy drugs cisplatin or docetaxel are the primary choice for these patients. However, most patients ultimately experience drug resistance or severe side effects^7^,^8^. In the present study, LCA obviously increased cell viability decrease, LDH release, and apoptosis in EGFR wild type NSCLC A549 and NCI-H1299 cells but not in normal cells (Figs 1 and 2). Besides, LCA is part of the chalcone class of compounds and the conventional template of these compounds allows for easy chemical modification, which results in obvious alterations in their biological functions and molecular targets^33^. Therefore, LCA might be a promising chemotherapeutics or chemical scaffold of chemotherapeutics for EGFR wild type NSCLC patients.

Previous studies indicated that LCA induced autophagy in prostate cancer LNCaP cells as evidenced by the increasing of the LC3-II expression and autophagosome accumulation^34^. However, up-regulation of LC3-II expression or autophagosome accumulation might be an autophagic inducer (increasing the autophagic flux) or inhibitor (suppressing the fusion of autophagosome with lysosome or the function of lysosome)^27^,^28^. Other methods such as combined treatment with CQ or knock-down of ATG proteins might be considered to detect this property^28^. The expression of CQ-accumulated LC3-II can be obviously increased when combined treatment of an autophagic inducer^35^, while an autophagic inhibitor could not increase CQ-accumulated LC3-II expression^36^. In the present study, the LC3-II expression in combined LCA and CQ treatment was more apparent than that in either LCA or CQ treated groups (Fig. 3B). Knock-down of ATG7 or ATG5, which are critical for autophagic progress^23^, also decreased LCA-induced LC3-II expression and GFP-LC3 punta formation (Fig. 3C,D). These studies confirmed that LCA could induce autophagy in NSCLC cells. However, it seems that inducing or inhibiting autophagic flux for a certain compound is relaying on the cell type, for instance, matrine suppressed autophagic flux in gastric cancer cells while increased autophagic flux in hepatocellular carcinoma cells^37^,^38^.

The effects of autophagy in cancer therapy are controversial^39^. Most studies indicated that autophagy can promote tumor survival but some agents-induced autophagy mainly contributes to cell death^40^,^41^. In the current study, inhibition of autophagy by pretreatment with CQ or silence of ATG7 could not change the LCA-induced cell viability and apoptosis in NSCLC cells (Fig. 4), suggesting LCA-decreased cell viability accompanied with autophagy. Induction of cell death accompanied with autophagy is also observed in other compounds. For example, inhibition of autophagy by pretreatment with CQ or siRNA of LC3 could not reverse dimethoxycurcumin-decreased cell viability^42^. However, LCA-induced autophagy in cervical cancer cells is cytoprotective^16^. This difference might be correlated with the autophagy-degraded cargo, *e.g.* tumor necrosis factor-related apoptosis-inducing ligand induced cytoprotective autophagy in HCT116 cells by the sequestration of the large caspase-8 subunit in autophagosomes and its subsequent elimination in lysosomes^43^. However, Fas induced pro-death autophagy through degradation of the tyrosine phosphatase Fas-associated phosphatase 1, which can dephosphorylate Fas and then reduce cell surface expression and activity of Fas, in autolysosome^44^.

CHOP, also known as growth arrest- and DNA damage-inducible gene 153, is one of the most critical components in the network of ER stress and plays critical roles in numerous diseases^45^. Some studies indicated that the activation of CHOP could decrease cell viability and increase apoptosis^46^,^47^, *e.g.* amino acid could decrease cell viability and increase apoptosis by CHOP activation^47^. Herein, we confirmed that CHOP was critical for LCA-decreased cell viability and -increased apoptosis. Besides, CHOP could bind to the promoters of ATG7, ATG5, and ATG10 *etc.*, and enhancing the expression of these ATGs to promote the autophagic process^47^,^48^. The effects of autophagy could promote cancer cell survival or death. Apoptosis-stimulating protein of p53-2, which binds to p53 to stimulate the transactivation function of p53 on the promoters of pro-apoptotic genes, could induce pro-death autophagy in hepatoma cells by activation of CHOP, while bufalin induced cytoprotective autophagy in glioma cells through CHOP activation^49^,^50^. In the present study, LCA induced autophagy by activation of CHOP, while the effects of autophagy were not involved in LCA-induced cell viability decrease or apoptosis. Thus, it is suggested that CHOP-induced autophagy might be pro-death, pro-survival, or accompanied depending on the stimulus.

**Figure 6:**
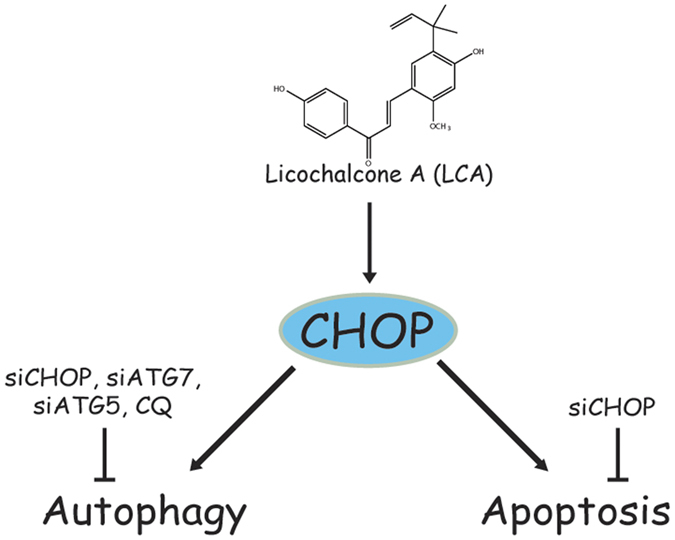
Schematic of LCA-induced apoptosis and autophagy in NSCLC cells. LCA induced autophagy and apoptosis by activation of CHOP. Autophagy inhibition could not alter LCA-induced cell viability decrease and apoptosis.

In conclusion, we demonstrated that LCA induced an accompanied autophagy in NSCLC cells, and the activation of CHOP was essential for LCA-induced cell viability decrease, apoptosis, and autophagy (Fig. 6). LCA might be a promising chemotherapeutics or chemical scaffold of chemotherapeutics for EGFR wild type NSCLC patients.

## Materials and Methods

### Reagents

LCA was purchased from National Institutes for Food and Drug Control (Beijing, China) and dissolved in dimethyl sulfoxide (DMSO) at a concentration of 40 mM, stored at −20 °C. MTT, CQ, G418, and DMSO were obtained from Sigma (St. Louis, MO, USA). 4′,6-diamidino-2-phenylindole (DAPI) were purchased from Beyotime Biotechnology Corporation–Shanghai (Shanghai, China). Dulbecco’s modified Eagle’s medium (DMEM) medium, RPMI 1640 medium, fetal bovine serum (FBS), penicillin, streptomycinwere, phosphate-buffered saline (PBS), and PI obtained from Gibco Life Technologies (Grand Island, NY, USA). GFP-LC3 (supplied by Toren Finkel, Addgene plasmid #24920)^51^ were obtained from Addgene. The primary antibodies, *i.e.* PARP, cleaved-caspase 7, cleaved-caspase 3, LC3, ATG7, ATG5, CHOP, BIP, GAPDH, and the responsive secondary antibodies were obtained from Cell Signaling Technology Inc. (Beverly, MA, USA).

### Cell line and culture

A549 and NCI-H1299 cells were obtained from the American Type Culture Collection (ATCC, Rockville, MD, USA). The A549 and NCI-H1299 cells were cultured in a RPMI 1640 medium supplemented with 10% (v/v) FBS and antibiotics (100 units/mL penicillin and 100 μg/mL streptomycin). HELF cells were purchased from Nanjing KeyGen Biotech Co. Ltd. (Nanjing, Jiangsu, China). HELF and HeLa cells with GFP-LC3 stable expression were cultured in a DMEM medium supplemented with 10% (v/v) FBS and 1% (v/v) antibiotics (100 units/mL penicillin and 100 μg/mL streptomycin). All cells were grown in a 5% CO_2_ incubator at 37 °C.

### MTT assay

The effects of LCA on cell viability were examined by MTT assay as described in the previous report^35^. Briefly, exponentially growing cells were seeded into 96-well plates. Upon reaching approximately 70% to 80% confluence, cells were treated as indicated. Then, the cell viability was detected by incubating the cells in a medium containing 1 mg/mL MTT for 4 h. 100 μL of DMSO was then added into solubilize the formazan and shaking for 10 min in the dark. The absorbance at 570 nm was recorded with a microplate reader (Perkin Elmer, 1420 Multilabel Counter Victor3, Wellesley, MA, USA).

### LDH assay

Cells were incubated to about 70–80% concentration on 96-well plates and then cultured with various concentrations of LCA for 24 h. The cellular toxicity was evaluated by study of LDH released into the cultured medium by the cytotoxicity detection kit strictly according to the manufacturer’s instructions (Beyotime Biotechnology Corp, Shanghai, China).

### PI staining assay

Cells seeded into 6-well plates were incubated with indicated concentrations of LCA for 24 h. Cells were harvested, washed with PBS, as well as fixed in 70% ethanol and stored at 4 °C overnight. Cells were then stained in PBS containing 5 μg/mL RNase and 20 μg/mL PI in the dark at room temperature for 30 min and analyzed using a flow cytometry (Becton Dickinson FACS Canto, Franklin Lakes, NJ). 10,000 events were recorded for each sample. The DNA content was analyzed using ModFit 161 LT version 3.0 software (Verity Software House, Topsham, USA).

### Western blot assay

After cells were treated with indicated compounds, total protein was extracted with a radioimmunoprecipitation lysis buffer containing 1% phenylmethanesulfonyl fluoride and 1% protease inhibitor cocktail for 20 min. The protein concentrations were determined with the BCA^TM^ protein assay kit (Pierce, Rockford, IL, USA). Equal amounts of proteins were separated by sodium dodecyl sulfate-polyacrylamide gel electrophoresis, and then transferred to a PVDF membrane followed by blocking in 5% non-fat dried milk in PBST at room temperature for 1 h. The membrane was incubated with specific primary antibodies overnight at 4 °C. After washing with PBST three times for 5 min each, the membranes were incubated with corresponding secondary antibodies at room temperature for 1 h. The specific protein bands were visualized with an ECL advanced western blot analysis detection kit (BD Biosciences, Bedford, MA, USA).

### Caspase 3/7 activity assay

Caspase 3/7 activity assay kits (Cell Signaling Technology, Inc., Beverly, MA, USA) were utilized to study caspase activities in accordance with the manufacturer’s instructions. Cells were plated into 96-well plates and cultured for 24 h. Cells were incubated with indicated concentrations of LCA with or without pretreatment of CQ (5 μM, 1 h) or specific knock-down of ATG7. Cells were then lysed on ice for 5 min and caspase 3/7 assay reagent (200 μL) was added into each well and incubated for 1 h. Luminescence was detected using a microplate reader (Perkin Elmer, 1420 Multilabel Counter Victor3, Wellesley, MA, USA).

### Annexin V-FITC and PI staining assay

After incubated with 10 μΜ LCA with or without pretreatment of CQ (5 μΜ, 1 h) or specific knock-down of ATG7, cells were trypsinized, washed, and collected. Apoptotic cells were studied using an annexin V-FITC kit (Beyotime Biotechnology Corporation, Shanghai, China) in accordance with the protocol provided by the manufacturer. A total of 10,000 cells were collected and analyzed using a flow cytometer (FACS-Canto, BD Bioscience, USA).

### siRNA transfection assay

The specific target sequences of ATG7 (sense 5′-GGUCAAAGGACGAAGAUAATT-3′, antisense 5′-UUAUCUUCGUCCUUUGACCTT-3′), ATG5 (sense 5′-GACGUUGGUAACUGACAAATT-3′, antisense 5′-UUUGUCAGUUACCAACGUCTT-3′), CHOP (sense 5′-GUUUCCUGGUUCUCCCUUGGUCUUTT-3′, antisense 5′-AAGACCAAGGGAGAACCAGGAAACTT-3′), and the scrambled siRNA (sense 5′-UUCUCCGAACGUGUCACGUTT-3′, antisense 5′-ACGUGACACGUUCGGAGAATT-3′) were synthesized by GenePharma (Shanghai, China). After cells seeded into 6-well plates for overnight, cells were transfected with specific siRNA of ATG7, ATG5, CHOP, as well as scrambled siRNA using Lipofectamine™ 2000 transfection reagent (Invitrogen Corp., Carlsbad, CA, USA) in strict accordance with the manufacturer’s instructions. Then, cells were incubated with LCA for 24 h. The protein expression levels were determined by western blot and GFP-LC3 punta were detected by immunofluorescence staining.

### Immunofluorescence staining assay

A549 cells were transiently transfected with GFP-LC3 plasmid for 24 h with or without subsequent 24 h transfection with specific siRNAs (i.e., scrambled, ATG7, and CHOP siRNAs) using Lipofectamine™ 2000 transfection reagent (Invitrogen Corp., Carlsbad, CA, USA) in accordance with the manufacturer’s instructions. Cells were then treated with the 10 μM LCA for 24 h. After incubation, cells were fixed with 4% PFA, washed with PBS, and then incubated with DAPI for 15 min. Immunofluorescence images were obtained by using a confocal laser scanning microscope (Leica TCS SP8, Solms, Germany).

### Statistical analysis

All experiments were repeated at least three times. The mean ± standard deviation (SD) was determined for each group. Statistical analysis was performed with one-way analysis of variance (one-way ANOVA) and Tukey’s test. Differences were considered statistically significant for (*) *P* < 0.05 and (**) *P* < 0.01. The “ns” means “no statistical difference”.

## Additional Information

**How to cite this article**: Tang, Z.-H. *et al.* Induction of C/EBP homologous protein-mediated apoptosis and autophagy by licochalcone A in non-small cell lung cancer cells. *Sci. Rep.* **6**, 26241; doi: 10.1038/srep26241 (2016).

## Supplementary Material

Supplementary Information
