# Supplementary material for: Induction of C/EBP homologous protein-mediated apoptosis and autophagy by licochalcone A in non-small cell lung cancer cells
Source: Sci Rep. 2016 May 17;6:26241. doi: 10.1038/srep26241 (PMC4869105; doi:10.1038/srep26241)

Induction of C/EBP homologous protein-mediated apoptosis and autophagy by licochalcone A in non-small cell lung cancer cells

Zheng-Hai Tang[1], Xin Chen[1], Zhao-Yu Wang[1], Ke Chai[1], Ya-Fang Wang[1], Xiao-Huang Xu[1], Xiao-Wen Wang[2], Jia-Hong Lu[1], Yi-Tao Wang[1], Xiu-Ping Chen[1], Jin-Jian Lu[1*]

Supplementary Information:

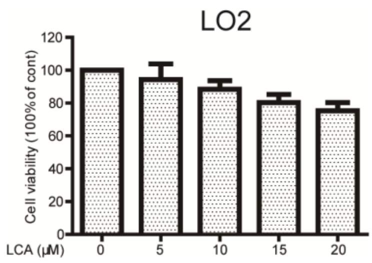

Figure S1. LCA presented no cytotoxicity in LO2 cells. LO2 cells were treated with indicated concentrations of LCA for 24 h. The cell viability was evaluated by MTT assay. **P* < 0.05, ***P* < 0.01, compared with 0 μM LCA treatment.

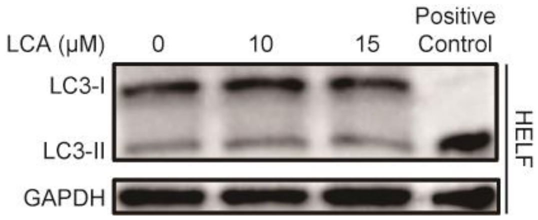

Figure S2. LCA could not induce LC3-II expression in HELF cells. HELF cells were treated with various concentrations of LCA for 24 h, and the cell extracts were analyzed for protein expression by western blot analysis.

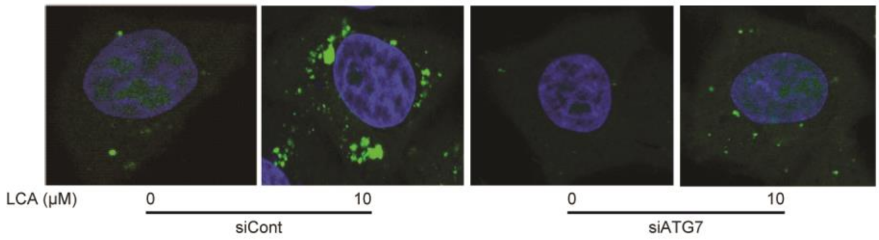

Figure S3. LCA induced GFP-LC3 punta formation in an ATG7-dependent manner. HeLa cells with GFP-LC3 stable expression were transiently transfected with ATG7 siRNA for 24 h. Cells were then treated with 10 μM LCA for 24 h and the GFP-LC3 punta formation was imaged using a confocal microscope.

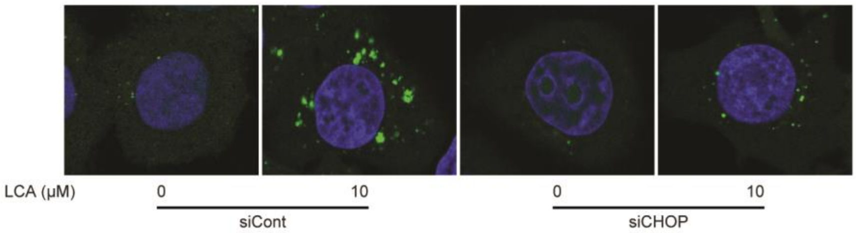

Figure S4. LCA induced GFP-LC3 punta formation in a CHOP-dependent manner. HeLa cells with GFP-LC3 stable expression were transiently transfected with CHOP siRNA for 24 h. Cells were then treated with LCA for 24 h and the GFP-LC3 punta formation was imaged using a confocal microscope.

The original data of western blot assay:

The results in the red block were presented in the manuscript.

Fig. 2B

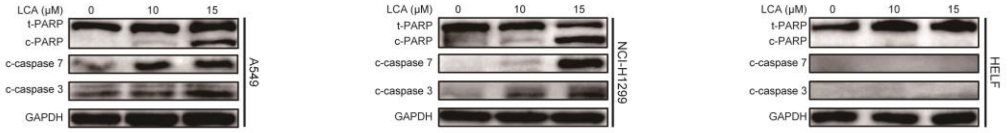

Original data of Fig. 2B

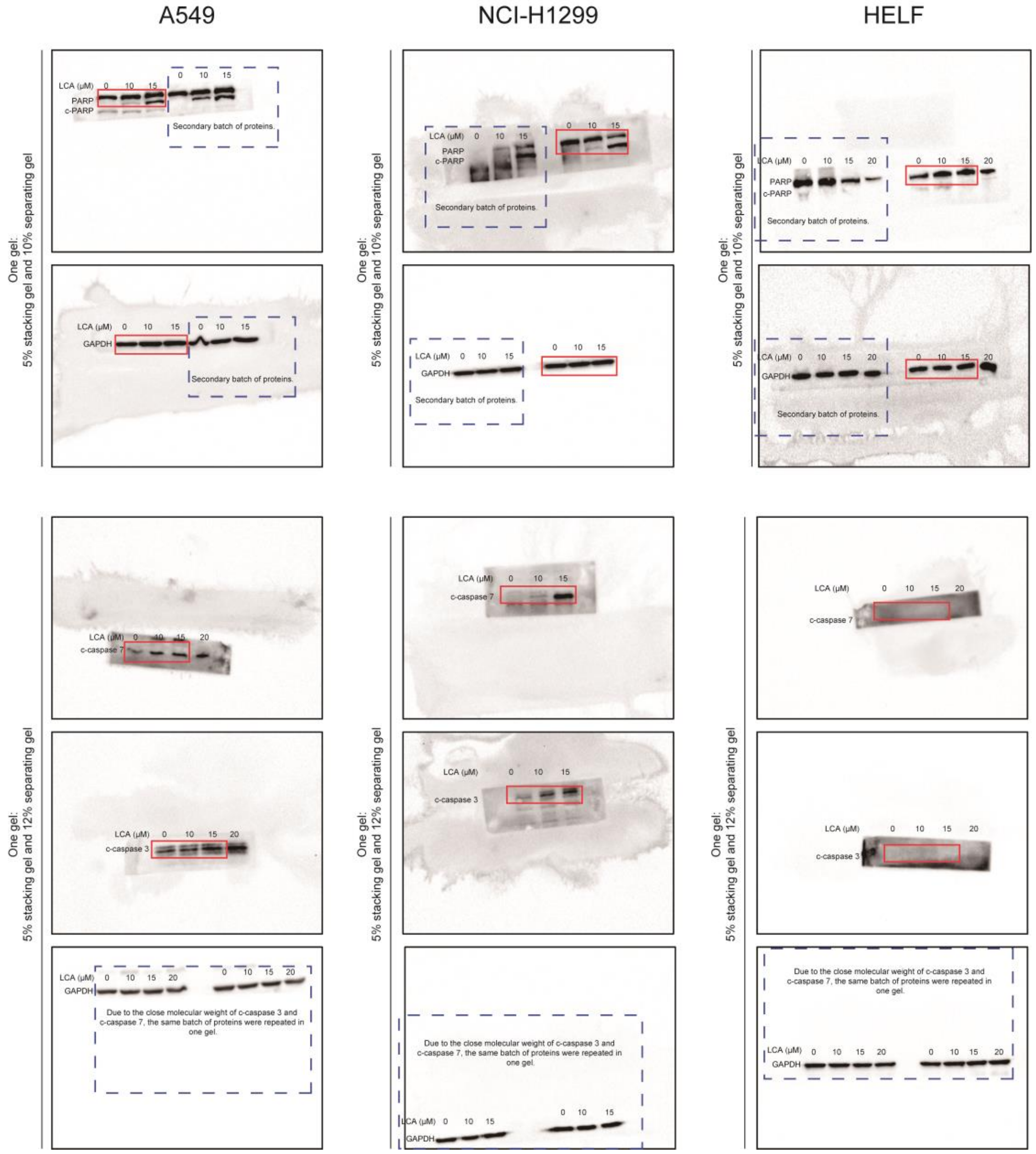

Fig. 3A

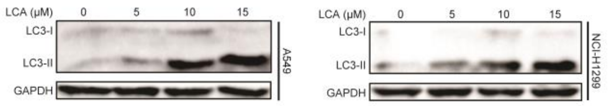

Fig. 3B

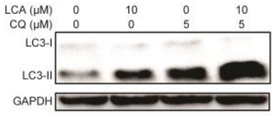

Original data of Fig. 3A

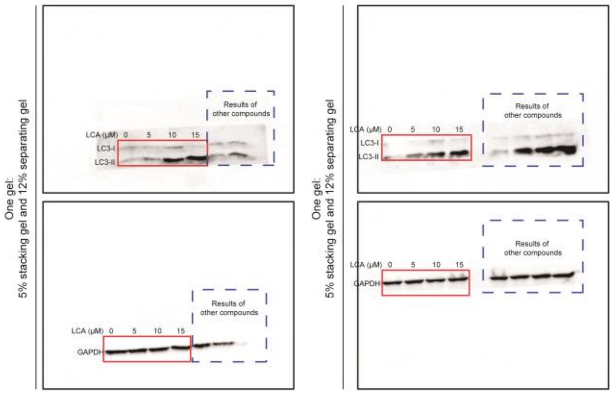

Original data of Fig. 3B

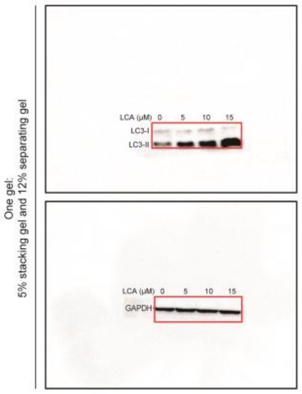

Fig. 3C

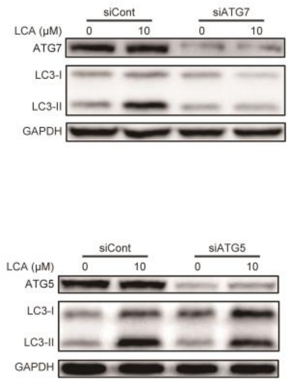

Original data of Fig. 3C

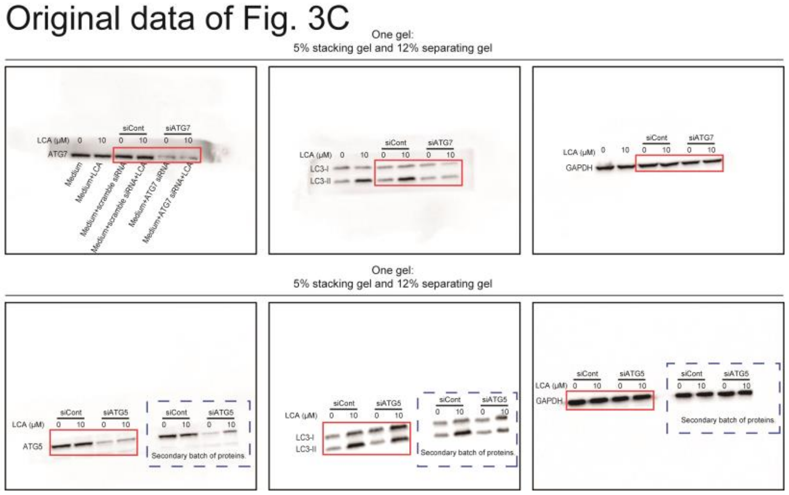

Fig. 5A

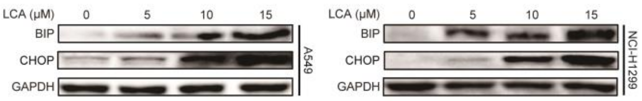

Fig. 5E

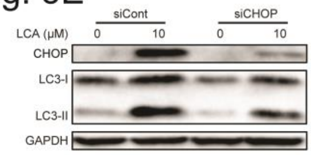

Original data of Fig. 5A

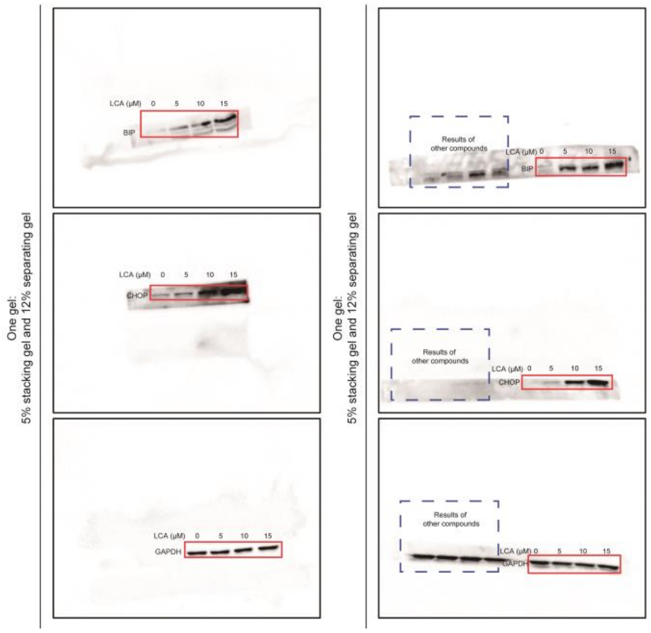

Original data of Fig. 5E

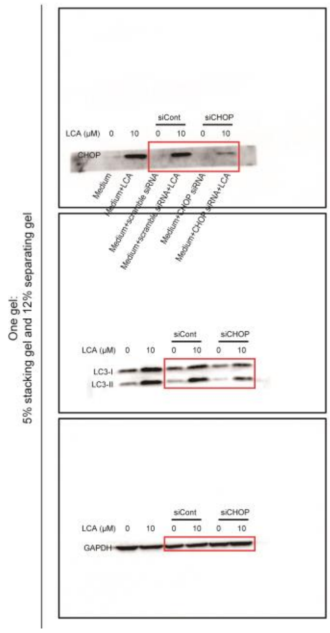

Supplement: Supplementary Information [file srep26241-s1.pdf]
